# Whole picture of human stratum corneum ceramides, including the chain-length diversity of long-chain bases

**DOI:** 10.1016/j.jlr.2022.100235

**Published:** 2022-05-30

**Authors:** Madoka Suzuki, Yusuke Ohno, Akio Kihara

**Affiliations:** Laboratory of Biochemistry, Faculty of Pharmaceutical Sciences, Hokkaido University, Sapporo, Japan

**Keywords:** ceramide, epidermis, lipidomics, long-chain base, mass spectrometry, skin barrier, sphingolipid, stratum corneum, A, α-hydroxy, CERS, ceramide synthase, d, di, *d*_9_, nine deuterium atoms, DS, dihydrosphingosine, EO, esterified ω-hydroxy, H, 6-hydroxysphingosine, HA, hemagglutinin, HEK, human embryonic kidney, KDS, 3-ketodihydrosphingosine, LCB, long-chain base, MRM, multiple reaction monitoring, N, nonhydroxy, O, ω-hydroxy, P, phytosphingosine, P-O, protein-bound ω-hydroxy, S, sphingosine, SC, stratum corneum, SD, 4,14-sphingadiene, SPT, serine palmitoyltransferase, t, tri

## Abstract

Ceramides are essential lipids for skin permeability barrier function, and a wide variety of ceramide species exist in the stratum corneum (SC). Although ceramides with long-chain bases (LCBs) of various lengths have been identified in the human SC, a quantitative analysis that distinguishes ceramide species with different LCB chain lengths has not been yet published. Therefore, the whole picture of human SC ceramides remains unclear. Here, we conducted LC/MS/MS analyses to detect individual ceramide species differing in both the LCB and FA chain lengths and quantified 1,327 unbound ceramides and 254 protein-bound ceramides: the largest number of ceramide species reported to date. Ceramides containing an LCB whose chain length was C16–26 were present in the human SC. Of these, C18 (28.6%) was the most abundant, followed by C20 (24.8%) and C22 (12.8%). Each ceramide class had a characteristic distribution of LCB chain lengths and was divided into five groups according to this distribution. There was almost no difference in FA composition between the ceramide species containing LCBs of different chain lengths. Furthermore, we demonstrated that one of the serine palmitoyltransferase (SPT) complexes, SPTLC1/SPTLC3/SPTSSB, was able to produce C16–24 LCBs. The expression levels of all subunits constituting the SPT complexes increased during keratinocyte differentiation, resulting in the observed chain-length diversity of LCBs in the human SC. This study provides a molecular basis for elucidating human SC ceramide diversity and the pathogenesis of skin disorders.

One of the most important functions of the skin is to provide a permeability barrier (skin barrier) that protects against the invasion of external pathogens, allergens, and chemical substances and prevents the loss of water and electrolytes from the body. Reduced skin barrier function increases the risk of or causes infectious diseases, atopic dermatitis, xerosis, and ichthyosis (1, 2, 3, 4). The lipid lamella, a multilayered lipid structure, is a constituent of the stratum corneum (SC; the outermost layer of the epidermis) and plays a central role in skin barrier function (5). Lipid lamellae are primarily composed of ceramides, cholesterol, and free FAs (6). Various ceramide species exist in the human SC, and a decrease in ceramide levels and/or a change in ceramide composition is associated with atopic dermatitis and psoriasis (7, 8, 9).

Ceramides consist of a long-chain base (LCB) to which an FA is attached via an amide bond. Mammals have five types of LCBs—dihydrosphingosine (DS), sphingosine (S), phytosphingosine (P), 6-hydroxysphingosine (H), and 4,14-sphingadiene (SD)—which differ in the number and position of their hydroxyl group and double bond(s) (Fig. 1A) (12, 13). LCBs can also be represented using the number of hydroxyl groups (d, di/2; t, tri/3), carbon atoms, and double bonds. For example, the five LCB types, which have a carbon chain length of 18, are designated as d18:0 (DS), d18:1 (S), t18:0 (P), t18:1 (6OH) (H), and d18:2 (SD), respectively (hereafter, we use d, t, or d/t as a prefix before the chain length to represent LCBs and C to represent FAs). The FA in the ceramide can also be categorized into four types in humans: nonhydroxy (N), α-hydroxy (A), ω-hydroxy (O), and esterified ω-hydroxy (EO) FAs (Fig. 1B) (12). The nomenclature recommended by LIPID MAPS (https://www.lipidmaps.org) is as follows: N FA, FA, or 16:0; A FA, FA (2OH) or 16:0 (2OH); O FA, FA (30OH) or 30:0 (30OH); and EO FA, ω-linoleoyloxy FA or ω-linoleoyloxy 30:0 (as illustrative examples, these numbers are correct for C16:0 [N and A] or C30:0 [O and EO] species). EO-type ceramides are referred to as acylceramides, and the predominant FA species that is esterified to its ω-position is linoleic acid (14). Human ceramides are classified into 20 classes based on their combination of LCB and FA. Each ceramide class is represented using the abbreviations of its type of FA and LCB (Fig. 1C). For example, the ceramide class composed of an N FA and S is denoted NS. In addition to these ceramide classes, SC contains protein-bound ceramides composed of an LCB and a protein-bound O FA (P-O). These protein-bound ceramides (P-O-type ceramides) are covalently attached to the surface proteins (cornified envelope proteins) of corneocytes, which are terminally differentiated keratinocytes (10, 15, 16, 17). There are five classes of protein-bound ceramides, classified according to the type of LCB they contain (P-ODS, P-OS, P-OP, P-OH, and P-OSD). Each ceramide class includes various molecular species that differ in FA chain length and/or degree of unsaturation. In the human SC, the chain lengths of the FA moiety range mainly from C16 to C30 in N- and A-type ceramides and C30 to C36 in O-, EO-, and P-O-type ceramides (12, 18, 19, 20).

**Fig. 1:**
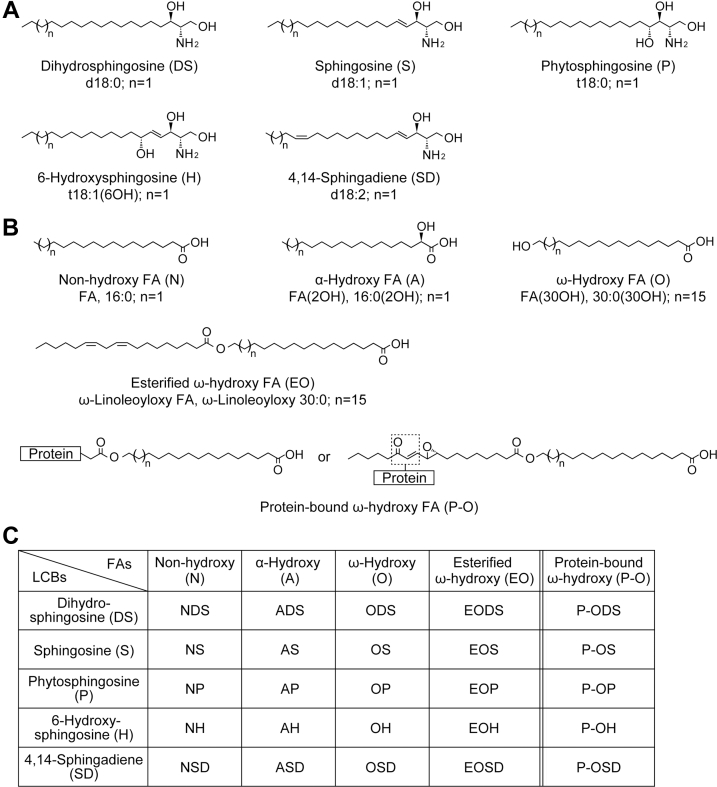
Structures and nomenclature of human ceramide classes. A and B: Structures of LCBs (A) and FAs (B) in human ceramides. The abbreviations used in this study are shown in parentheses after each compound name, and those recommended by LIPID MAPS (https://www.lipidmaps.org) are shown below them, with each number corresponding to an n value of 1 or 15, as indicated in the figure. Two models have been proposed for how protein-bound ceramides bind to corneocyte envelope proteins: one is that protein-bound ceramides bind to proteins via the ω-hydroxyl group of the FA moiety after the release of the modified linoleic acid moiety (10), and the other is that they bind to proteins via the enone of the modified linoleic acid moiety (dotted box) (11). C: Notation of ceramide classes. Ceramide classes are designated using a combination of the abbreviations for types of FA and LCB.

Ceramides are the hydrophobic backbone of sphingolipids, which are one of the major lipid components of cell membranes (12). In most tissues, ceramide levels are low since ceramides are transiently produced only during the synthesis and degradation of sphingolipids. Furthermore, only a limited number of classes of ceramides exist in most mammalian tissues; NS is the predominant ceramide class, and other classes are present at low levels or only in specific tissues. By contrast, SC contains high levels and multiple classes of ceramides, which enables it to perform its specialized role, namely, skin barrier function. In human SC, most unbound ceramide classes and all five protein-bound ceramide classes are present (18, 19, 20). Among these, acylceramides (EO-type ceramides) and protein-bound ceramides (P-O-type ceramides) are crucial for skin barrier formation. Acylceramides are involved in the formation and maintenance of lipid lamellae (21, 22). Protein-bound ceramides form the plasma membrane-like structure of corneocytes, known as the corneocyte lipid envelope, and are considered to function as a scaffold for the lipid lamellae (15, 17). Mutations in any of the genes involved in the production of acylceramides or protein-bound ceramides cause congenital ichthyosis (23, 24, 25). Furthermore, mice lacking one of these genes exhibit impaired skin barrier function and die shortly after birth (11, 26, 27, 28, 29, 30, 31, 32, 33).

Although sphingolipids containing d/t18 LCBs are predominant in most mammalian tissues, those with LCBs other than d/t18 are also found in several tissues. For example, gangliosides (glycosphingolipids containing one or more sialic acids) with a d20:1 LCB have been found in the brain, stomach, and intestinal mucosa (34, 35), and sphingomyelins with d16–20 LCBs are present in plasma (36). However, the chain length of the most abundant LCB in these tissues is d18. Serine palmitoyltransferase (SPT) catalyzes the condensation reaction of serine with acyl-CoA—the initial step in de novo sphingolipid synthesis—to produce 3-ketodihydrosphingosine (KDS) (12, 37). Palmitoyl-CoA is used as a substrate to generate C18 KDS, which is a precursor of d/t18 LCBs. Mammalian SPT is a heterotrimer composed of two large subunits (one is necessarily SPTLC1 and the other is either SPTLC2 or SPTLC3) and one of two small subunits (SPTSSA or SPTSSB) (37, 38). Therefore, SPTLC1/2/SSA, SPTLC1/3/SSA, SPTLC1/2/SSB, and SPTLC1/3/SSB are the four types of mammalian SPT complexes. SPTLC2 and SPTLC3 are catalytic subunits that bind to the coenzyme pyridoxal 5′-phosphate (37, 39). SPTLC1 is required for the stability of SPTLC2 (40) and may also be for SPTLC3. The small subunits confer full enzyme activity to the large subunits and modulate the substrate specificities of SPTs (38). Each SPT complex exhibits different substrate specificities toward acyl-CoAs: SPTLC1/2/SSA, C16:0-CoA; SPTLC1/2/SSB, C16:0-CoA and C18:0-CoA; SPTLC1/3/SSA, C14:0-CoA and C16:0-CoA; and SPTLC1/3/SSB, C14:0–C20:0-CoA (38, 41, 42). However, the SPT complex that is involved in the production of d/t24 or longer LCBs has not yet been identified.

Epidermal ceramide analyses were initially performed using TLC and GC (43, 44). Then, especially in the last 2 decades, MS and MS/MS were developed for the analysis of sphingolipids and ceramides, and these methods are superior in specificity, quantitation, and sensitivity (45). In MS(/MS), lipid separation was often combined with LC, and the LC/MS(/MS) technique is particularly useful in the analysis of diverse epidermal ceramides. LC/MS/MS analyses of ceramides using the product ion scanning mode, as well as GC/MS analysis of LCBs generated from ceramides by acid-catalyzed methanolysis, revealed the presence of various ceramide/LCB classes and species in the human SC (19, 20, 46, 47, 48). In the product ion scanning mode of LC/MS/MS, the molecular ions with specific mass-to-charge ratio (*m/z*) values that correspond to specific ceramide species are selected as precursor ions and fragmented via collision-induced dissociation. Based on the *m/z* values of the product ions generated, the LCB class and chain length are determined. This scanning mode has the advantage of allowing the discovery of unexpected molecular species since the product ions are not selected by setting the procedure to detect specific *m/z* values but rather a range of *m/z* values. However, it is difficult to detect small quantities of molecules using this scanning mode because of the low sensitivity of this method, and the optimal collision voltage cannot be applied to individual molecular species in this mode. Accordingly, this scanning mode is generally used for qualitative analysis and is not suitable for quantitative analysis. In addition, it is impractical to subject the large number of ceramide species present in the SC to product ion scanning analysis to identify all the ceramide classes and LCB chain lengths. Ceramide species in the human SC have previously been quantified using LC/MS analysis (5, 20, 49, 50). However, this method can only specify the total chain length of both the FA and the LCB combined, and it is technically impossible to determine the individual chain lengths of the two moieties. LC/MS/MS analysis using the multiple reaction monitoring (MRM) mode is a highly sensitive and quantitative method, since optimized settings such as the collision energies and the *m/z* values of the precursor and product ions can be applied when analyzing individual molecular species of interest. Moreover, MRM mode allows the detection and quantification of multiple molecular species in a single measurement. Therefore, MRM mode is useful for uncovering the whole picture of SC ceramides, including the total number and quantity of ceramide species with LCBs of different chain lengths. Recently, we established a highly accurate and sensitive method, using the MRM mode of LC/MS/MS and various ceramide class standards, which enables comprehensive quantification of ceramide species that belong to different ceramide classes and have different FA chain lengths (18). Using this method, we successfully identified and quantified unbound ceramides (345 species) and protein-bound ceramides (63 species) with d/t18 LCBs in the human SC. However, those with chain lengths other than d/t18 have not yet been numerated or quantified, meaning that the whole picture of human SC ceramides remains unclear. In the present study, we expanded our previously established method to quantify ceramide species with d/t16–26 LCBs and thus clarified the whole picture of the composition of FAs and LCBs constituting the human SC ceramide classes. In addition, through an SPT activity assay using mammalian cells and an expression analysis of SPT subunits in differentiated keratinocytes, we revealed that the chain-length diversity of LCBs is created by the high expression of all SPT subunits in the human SC.

## Materials and methods

### Ethics

This study was approved by the ethics committee of Hokkaido University and conducted in accordance with the ethical principles of the Declaration of Helsinki. Informed consent was obtained from all participants.

### Cell culture and transfection

Human embryonic kidney (HEK) 293T cells were cultured in DMEM (D6429; Merck, Darmstadt, Germany) supplemented with 10% FBS (Thermo Fisher Scientific, Waltham, MA), 100 units/ml penicillin, and 100 μg/ml streptomycin (Merck). Human primary epidermal keratinocytes (CELLnTEC, Bern, Switzerland) were cultured in CnT-Prime Epithelial Culture Medium (CELLnTEC). Differentiation was induced by replacing the medium with CnT-Prime 3D Barrier Medium (CELLnTEC) when the cells reached 100% confluence. The cells were cultured for 7 days, and the medium was refreshed every 3 days. Cells were grown on collagen-coated dishes (Iwaki, Shizuoka, Japan) and cultured at 37°C under 5% CO_2_. Transfections were performed using Lipofectamine Transfection Reagent with PLUS Reagent (Thermo Fisher Scientific) according to the manufacturer’s instructions.

### Plasmids

Human *SPTLC1*, *SPTLC2*, *SPTLC3*, *SPTSSA*, and *SPTSSB* genes were amplified by PCR using complementary DNA prepared from human primary keratinocytes (differentiated for 7 days) and their respective forward (-F) and reverse (-R) primers (supplemental Table S1). The amplified DNAs were first cloned into pGEM-T Easy vector (Promega, Madison, WI). After sequence confirmation, each gene was transferred to the mammalian expression vector pCE-puro-3×FLAG-1 or pCE-puro hemagglutinin (HA)-1, which are designed to produce N-terminal 3×FLAG-tagged or HA-tagged proteins, respectively (51). The *SPTLC1*, *SPTSSA*, and *SPTSSB* genes were introduced into pCE-puro 3×FLAG-1 vector, and the *SPTLC2* and *SPTLC3* genes were cloned into pCE-puro HA-1 vector.

### Lipid analyses via LC/MS/MS

Human SC samples were collected via tape stripping from healthy volunteers (seven males and three females aged 20–30 years). Unbound ceramides were extracted from the tape strips, which had sampled the human SC, as described previously (18). To quantify the ceramides, we used the following ceramides, each containing nine deuterium atoms (*d*_9_), as internal standards: *N*-palmitoyl(*d*_9_)-d-*erythro*-S (d18:1/*d*_9_-C16:0 NS), *N*-palmitoyl(*d*_9_)-DS (d18:0/*d*_9_-C16:0 NDS), *N*-palmitoyl(*d*_9_)-d-*ribo*-P (t18:0/*d*_9_-C16:0 NP), *N*-palmitoyl(*d*_9_)-6-(*R*)-H (t18:1(6OH)/*d*_9_-C16:0 NH), *N*-(2'-(*R*)-hydroxypalmitoyl(*d*_9_))-d-*erythro*-S (d18:1/*d*_9_-C16:0 AS), *N*-(2'-(*R*)-hydroxypalmitoyl(*d*_9_))-d-*erythro*-DS (d18:0/*d*_9_-C16:0 ADS), *N*-(2'-(*R*)-hydroxypalmitoyl(*d*_9_))-d-*ribo*-P (t18:0/*d*_9_-C16:0 AP), and *N*-(2'-(*R*)-hydroxypalmitoyl(*d*_9_))-6-(*R*)-H (t18:1(6OH)/*d*_9_-C16:0 AH) (all purchased from Avanti Polar Lipids, Alabaster, AL). Protein-bound ceramides were extracted essentially as described previously (18). After extensive washing to remove unbound lipids from the SC samples, internal standards (d18:1/*d*_9_-C16:0 AS, d18:0/*d*_9_-C16:0 ADS, t18:0/*d*_9_-C16:0 AP, and t18:1(6OH)/*d*_9_-C16:0 AH) were added. The SC samples were treated with alkaline to release protein-bound ceramides as O-type ceramides.

Ceramides were quantified using an ultra-performance LC-coupled triple quadruple mass spectrometer (Xevo TQ-S; Waters, Milford, MA). The LC conditions were essentially as previously described (52). Briefly, the separation was achieved on a reverse-phase column (ACQUITY UPLC CSH C18 column; length 100 mm; particle size 1.7 μm; inner diameter 2.1 mm; Waters) using a binary gradient with mobile phase A (acetonitrile/water [3:2, v/v] containing 5 mM ammonium formate) and mobile phase B (2-propanol/acetonitrile [9:1, v/v] containing 5 mM ammonium formate). Ionization was performed via electrospray ionization in the positive ion mode using the following settings: capillary voltage, 2.5 kV; cone voltage, 30 V (for N-, A-, and O-type ceramides) or 46 V (EO-type ceramides); source temperature, 140°C; desolvation temperature 650°C; desolvation gas flow, 1,200 l/h; and nebulizer gas, 7.0 bar. In the product ion scanning analysis, the *m/z* values and collision energy voltage were set as follows: NS46 (here and below, the number represents the sum of the LCB and FA chain lengths), 688.7, 30 eV; NS47, 702.7, 30 eV; NDS46, 708.7, 30 eV; NP46, 724.8, 35 eV; and NH46, 704.7, 30 eV. The *m/z* values of the precursor (Q1) and product (Q3) ions and the collision energies used in the MRM analysis are listed in supplemental Table S2. Since NS, AS, NH, AH, NSD, and ASD are mostly dehydrated during ionization via in-source decay (18), we only set the *m/z* values for the [M + H – H_2_O]^+^ for Q1 to detect these ceramide classes. In contrast, since similar quantities of nondehydrated and dehydrated forms are generated for EOS, OS, EOH, OH, EOSD, and OSD, we set the *m/z* values for both [M + H]^+^ and [M + H – H_2_O]^+^ for Q1. Since the DS- and P-type ceramides are ionized mostly as nondehydrated forms, we only set the *m/z* values for [M + H]^+^ to detect these ceramide classes. To detect ceramide species containing d/t18 LCB, we set the *m/z* values of the LCB type-specific product ions, which were verified using standards for each ceramide with a d/t18 LCB (18), for Q3. For ceramide species containing LCBs other than d/t18, we set the *m/z* values based on the difference in the number of carbon and hydrogen atoms from d/t18 LCBs for Q3. Although the LCB chain length did not affect the efficiency of the ceramide fragmentation by collision-induced dissociation, longer FA chain lengths did reduce it. We therefore optimized the collision energy settings based on product ion scanning and MRM analysis using each ceramide class-specific d/t18 ceramide species containing *d*_9_-C16:0 (ceramide standard), C24:0 (SC ceramide), or C30:0 (SC ceramide) FA. Ceramides were quantified by calculating the ratio of the peak area for each ceramide species to that of the internal standard (*d*_9_-labeled ceramide) corresponding to each ceramide class. Since *d*_9_-labeled SD-type ceramides are not commercially available, we quantified SD-type ceramides using *d*_9_-labeled S-type ceramide standards. O-, EO-, and P-O-type ceramides were quantified using the *d*_9_-labeled A-type ceramide standards that contain common LCB structures.

Lipid extractions from HEK 293T cells were performed as follows. Cells grown in a 12-well plate were washed twice with 1 ml PBS, recovered using a scraper, and transferred into plastic tubes. The cells were pelleted down by centrifugation (400 *g*, 4°C, 3 min), suspended in 100 μl H_2_O, and mixed with 375 μl chloroform/methanol (1:2, v/v) and 2.5 pmol d18:1/*d*_9_-C16:0 NS (internal standard). To hydrolyze the ester bond in the glycerolipids, the samples were treated with 25 μl 3 M KOH and incubated at 37°C for 1 h. After neutralization via the addition of 30 μl of 3 M formic acid, the samples were mixed with 125 μl of chloroform and 125 μl of water and centrifuged (20,400 *g*, room temperature, 3 min) to separate the phases. The lower (organic) phase was collected and dried. The dried lipids were dissolved in 125 μl of chloroform/methanol (1:2, v/v) and analyzed using LC/MS/MS (injection volume, 5 μl).

### Quantitative real-time RT-PCR

Human primary keratinocytes cultured in a 24-well plate were washed twice with 0.5 ml PBS. Total RNAs were recovered using a NucleoSpin RNA II Kit (Takara Bio, Shiga, Japan) according to the manufacturer’s protocols. Quantitative real-time RT-PCR was performed using a One-Step TB Green PrimeScript RT-PCR Kit II (Takara Bio) and forward (-F) and reverse (-R) primer pairs (supplemental Table S3).

## Results

### Clarification of the existence of diverse ceramide species via LC/MS/MS analyses

**Fig. 2:**
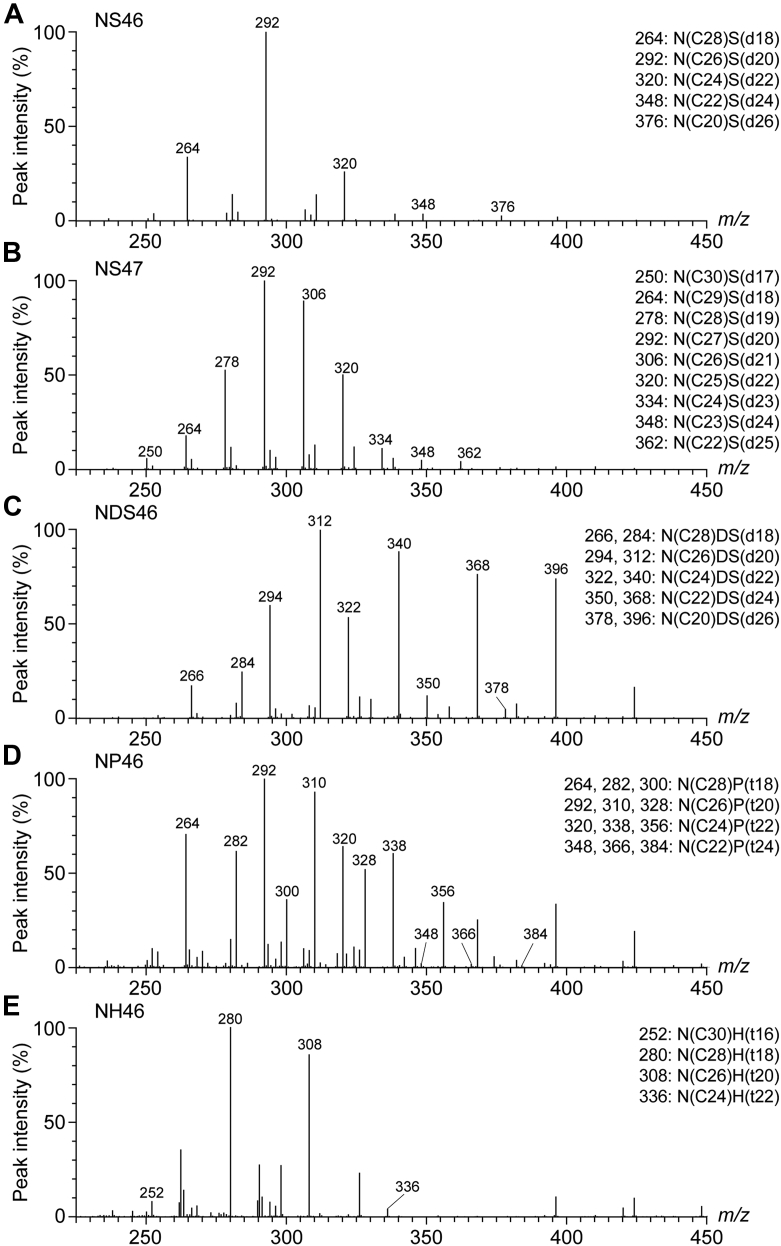
Qualitative analysis of the chain-length diversity of LCBs in human SC ceramides via product ion scanning. A–E: Lipids were extracted from human SC samples collected by tape stripping and subjected to LC/MS/MS analysis using the product ion scanning mode to detect NS46 (A), NS47 (B), NDS46 (C), NP46 (D), and NH46 (E) ceramides. The *y*-axis shows the relative value, with the highest peak as 100%. The number in each ceramide category (such as NS46) is the total chain length of the LCB and FA moieties combined. The values above each peak are the *m/z* values (nominal mass) for specific product ions of each ceramide species, and the corresponding ceramide species are listed on the right of each spectrum.

To investigate the distribution of the chain lengths of LCBs in human SC ceramides, we first performed an LC/MS/MS analysis using the product ion scanning mode. In a product ion scanning analysis of ceramides, the ceramide species are selected as precursor ions using the sum of the LCB and FA chain lengths. For example, the *m/z* value of the precursor ion [M + H – H_2_O]^+^ of NS with a total chain length of 46 (NS46) is 688, and this includes N(C30)S(d16), N(C28)S(d18), N(C26)S(d20), N(C24)S(d22), N(C22)S(d24), N(C20)S(d26), and more (the numbers in parentheses indicate the chain lengths of the FA [C] and the LCB [d]). Each type of LCB has a characteristic fragmentation pattern in the positive ion mode (18). S-type ceramides with a d18 LCB generate their predominant product ion at *m/z* 264. The lipids were extracted from human SC that was collected by tape stripping, and they were subjected to a product ion scanning analysis for NS46 ceramides. NS46 generated product ions at *m/z* 264, 292, 320, 348, and 376 (Fig. 2A), corresponding to the S-type specific product ions generated from N(C28)S(d18), N(C26)S(d20), N(C24)S(d22), N(C22)S(d24), and N(C20)S(d26), respectively. This indicates that NS species containing LCBs with d18–26 are present in human SC, which is consistent with past reports (19, 20). In addition to ceramide species containing even-chain FAs, species containing odd-chain FAs are also present in the human SC (18). Next, to investigate the presence of ceramides with odd-chain LCBs or FAs, we performed a product ion scanning analysis of NS47 (*m/z* value of 702, [M + H – H_2_O]^+^). NS47 yielded product ions at *m/z* 250, 264, 278, 292, 306, 320, 334, 348, and 362 (Fig. 2B), corresponding to the product ions of N(C30)S(d17), N(C29)S(d18), N(C28)S(d19), N(C27)S(d20), N(C26)S(d21), N(C25)S(d22), N(C24)S(d23), N(C23)S(d24), and N(C22)S(d25), respectively. These results indicate that NS species containing LCBs ranging from d17 to d26 exist in the human SC. We also performed a product ion scanning analysis of NDS, NP, and NH with total chain lengths of 46. We previously showed that these species, which contain d/t18 LCB, generate their main product ions at *m/z* 266 and 284 (NDS), 264, 282, and 300 (NP), and 280 (NH) (18). NDS46 yielded product ions corresponding to NDS species containing d18, d20, d22, d24, and d26 LCBs (Fig. 2C). Although ceramides with longer LCBs, such as d24 or d26, were detected only at low levels in the case of NS46 (Fig. 2A), NDS46 yielded relatively large peaks corresponding to d24 and d26 species (Fig. 2C). We also detected NP46 and NH46 species containing even-chain LCBs with t18–24 and t16–22, respectively (Fig. 2D, E). We thus demonstrated that ceramide species containing both even- and odd-chain LCBs with d/t16–26 are present in the human SC.

**Fig. 3:**
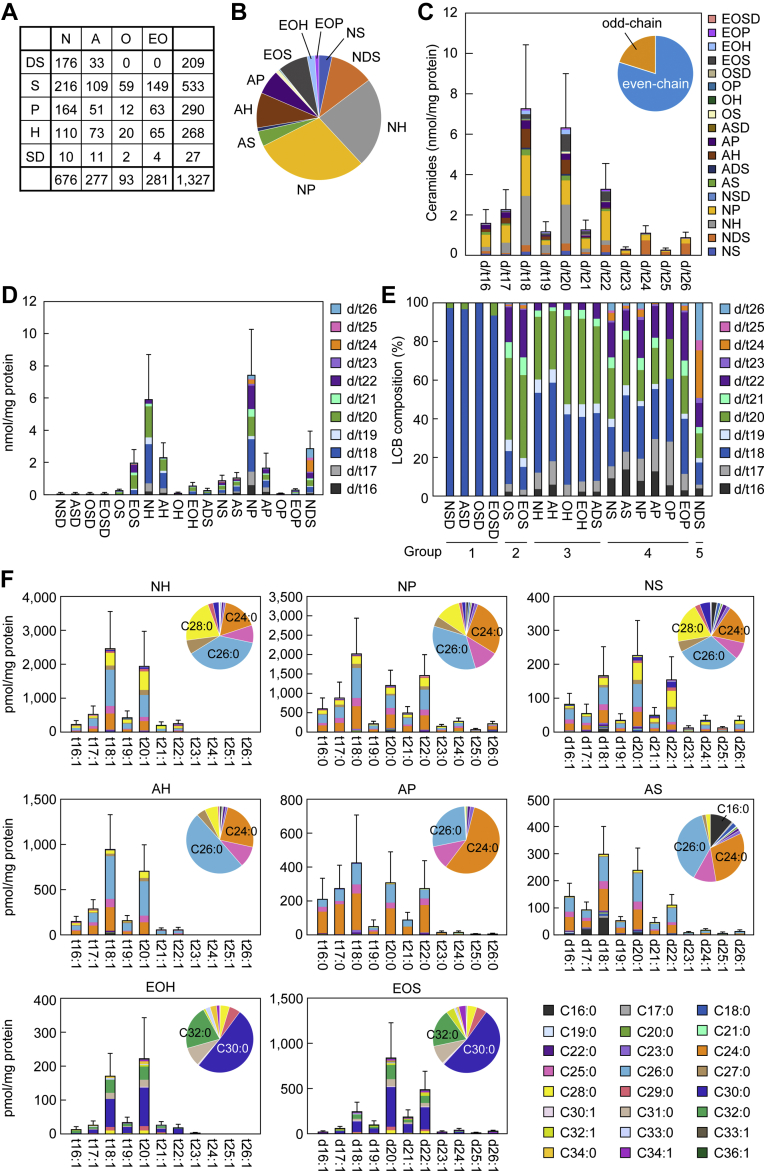
Diversity of unbound ceramides in the human SC. Human SC samples were collected by tape stripping (n = 10). Lipids were extracted from the tape and subjected to LC/MS/MS analysis in MRM mode. A: Numbers of ceramide species identified in each ceramide class. B: The proportion of the quantity of each ceramide class to the total quantity of ceramides. C: The total quantity of ceramides with each LCB chain length. Values are means + SDs of the total quantity of each LCB chain-length category, and each ceramide class is color coded. The pie chart shows the proportion of ceramides with even- and odd-chain LCBs out of total ceramides. D and E: The total quantity (D) and LCB chain-length composition (D and E) of each ceramide class. Values are means + SDs of the total quantity (D) and the proportion (E) of each LCB chain-length category of each ceramide class. Each ceramide is color coded according to its LCB chain length. The group numbers of the five groups of ceramide classes, classified based on the distribution pattern of LCB chain lengths, are shown below the graph (E). F: The total quantity of representative unbound ceramide species, categorized according to LCB chain length (indicated along the *x*-axis) and FA chain length (indicated by color coding). Values are means + SDs. The pie chart shows the ratio of ceramides with each FA chain length to the total for each ceramide class.

Product ion scanning analysis is less suitable for detecting small quantities of molecules, applying optimal detection settings for individual molecular species, and performing quantitative and comprehensive analysis. To address these problems and further elucidate the whole picture of ceramide species in the human SC that differ in LCB and FA chain length, we conducted an LC/MS/MS analysis using MRM mode to target 7,150 ceramide species. Although the LCB chain length did not affect the efficiency of fragmentation of the ceramides via collision-induced dissociation, longer FA chains did reduce it. We therefore optimized the collision energies, which were determined by measuring ceramide species with different chain lengths. We used internal ceramide standards consisting of *d*_9_-labeled C16:0 FA and d/t18 LCB for quantification. Since *d*_9_-labeled ceramides containing LCBs other than d/t18 are not commercially available, we quantified ceramide species containing those LCBs by calculating the ratio of the peak area for each ceramide species to that of the ceramide standard containing a d/t18 LCB corresponding to each ceramide class. We previously identified 345 ceramide species via MRM measurement focusing on those with d/t18 LCBs (18). Here, we identified 1,327 ceramide species by targeting ceramide species with different LCB chain lengths (Fig. 3A). The ceramide class containing the largest number of ceramide species was NS (216), followed by NDS (176), NP (164), and EOS (149). The most abundant ceramide class was NP (29.4%), followed by NH (23.4%), NDS (11.3%), AH (9.1%), and EOS (7.7%) (Fig. 3B and supplemental Table S4). Consistent with the results obtained in the product ion scanning analyses (Fig. 2), ceramide species containing LCBs ranging from d/t16 to d/t26 were present (Fig. 3C and supplemental Table S5). Among these, d/t18 was the most abundant (28.6%), followed by d/t20 (24.8%) and d/t22 (12.8%). Ceramide species containing odd-chain LCBs such as d/t17, d/t19, and d/t21 were also present at modest levels (8.9%, 4.5%, and 4.9%, respectively). Odd-chain LCBs accounted for 20.2% of the total ceramides.

Each ceramide class showed a characteristic LCB chain-length composition (Fig. 3D, E and supplemental Table S6), and the ceramide classes were classified into five groups according to their predominant LCB chain lengths: group 1, d/t18; group 2, d/t20; group 3, d/t18 and d/t20; group 4, d/t18, d/t20, and d/t22; and group 5, d/t18–26 (Fig. 3E). Group 1 ceramides included only SD-type ceramides (NSD, ASD, OSD, and EOSD), and d18 species accounted for more than 90% of these, with a trace amount of d20 species present. Group 2 ceramides comprised OS and EOS, in which d20 species were predominant (≥40%), followed by d22 (∼20%) and d18 (∼15%). H-type ceramides (NH, AH, OH, and EOH) and ADS belonged to group 3, and both d/t18 and d/t20 species accounted for ∼40% each. Ceramides with an LCB longer than d/t23 were below the detection limit. Group 4 ceramides included NS, AS, and P-type ceramides (NP, AP, OP, and EOP). In this group, d/t18-, d/t20-, and d/t22-containing ceramides were predominant, each of which accounted for 11–33%. Group 5 ceramides included only NDS, which were characterized by LCBs with a wide range of chain lengths (d16–26). Among these, d24 (24.5%) and d26 (19.2%) species were particularly abundant, although they were present at low levels in the other groups.

We previously demonstrated that N- and A-type ceramides with d/t18 LCBs are predominantly composed of C24–28 FAs, with either C24 or C26 the most abundant (18). An exception to this is C16:0 FA, which is also abundant, but only in AS. Here, we observed almost no difference in FA composition between the ceramides containing LCBs of different chain lengths, except in AS (Fig. 3F and supplemental Table S7). Consistent with our previous report (18), C16:0, C24:0, and C26:0 FAs were abundant in AS with d18 and also in AS with d17. By contrast, the proportion of C16:0 was low in AS containing LCBs other than d17 or d18. The principal FA chain lengths in acylceramides (EO-type ceramides) containing d/t18 LCBs are C30–32, with C30 the most abundant, as reported previously (18). The FA composition of acylceramides containing LCBs other than d/t18 was similar to those containing d/t18 LCBs. Note that the chain lengths and degree of unsaturation of the FAs in acylceramides can only be determined as the sum of the two FAs (the O [O-type] FA binding to the LCB and the FA binding to the O FA) in our LC/MS/MS analysis. Here, we calculated the chain length and degree of unsaturation of the O FA assuming that the latter FA is linoleic acid (C18:2), since that is the predominant FA that binds to the O FA moiety of acylceramides in the human SC (14). We thus revealed that diverse ceramide species containing LCBs and FAs of various chain lengths exist in the human SC, and that the LCB chain-length composition differs according to ceramide class.

### Quantification of protein-bound ceramide species containing LCBs of various chain lengths

**Fig. 4:**
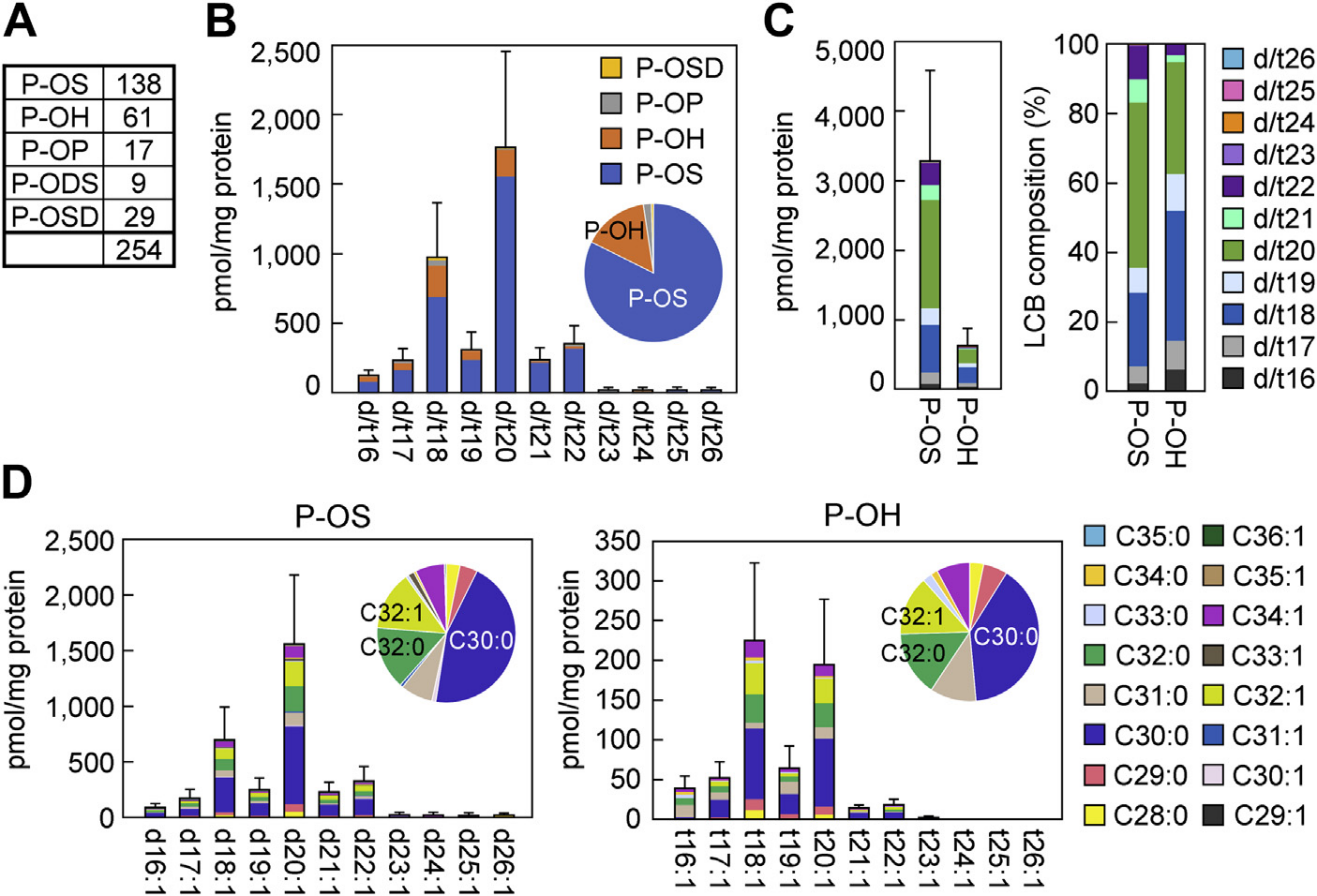
Diversity of protein-bound ceramides in the human SC. Human SC samples were collected by tape stripping (n = 10). Noncovalently bound lipids were removed from the SC samples, and the samples were treated with alkaline to release protein-bound ceramides (P-O-type ceramides) as O-type ceramides. These O-type ceramides were extracted and subjected to LC/MS/MS analysis in MRM mode. A: The number of ceramide species identified in each protein-bound ceramide class. B: The quantity of protein-bound ceramides with each LCB chain-length category (*x*-axis) color coded according to ceramide class. Values are means + SDs. The pie chart shows the ratio of each ceramide class to total protein-bound ceramides. C: Quantity and LCB chain-length composition of representative protein-bound ceramide classes. Values are means + SDs of the total quantity (left) and the proportion (right) of each LCB chain-length category of the protein-bound ceramide classes P-OS and P-OH. Ceramides are color coded according to LCB chain length. D: The total quantity of ceramides with each LCB chain length (*x*-axis) and FA chain length (color coding) in representative protein-bound ceramide classes. Values are means + SDs. The pie chart shows the ratio of protein-bound ceramides with each FA chain-length category to the total quantity of protein-bound ceramides in that class.

Although the precise structure of protein-bound ceramides (P-O-type ceramides) remains unclear, two structural models have been proposed: in one, the ceramide binds to the protein via an ester linkage involving the ω-hydroxy group of O FA moiety, which is exposed by the cleavage of the modified linoleic acid moiety (10); in the other, the ceramide binds to the protein via an unidentified covalent bond through the modified linoleic acid moiety (11) (Fig. 1B). To quantify protein-bound ceramides, the human SC samples were extensively washed with methanol to remove unbound ceramides, and protein-bound ceramides were then released as O-type ceramides by alkaline treatment. Both models of protein-bound ceramide would be converted to an O-type ceramide through this procedure, enabling us to quantify total protein-bound ceramide levels without considering the precise P-O-structure. We subjected the extracted O-type ceramides to MRM measurement and successfully identified 138, 61, 17, 9, and 29 protein-bound ceramide species (254 in total) of P-OS, P-OH, P-OP, P-ODS, and P-OSD, respectively (Fig. 4A). In terms of the total quantity of each protein-bound ceramide class, P-OS and P-OH were most abundant, accounting for 82.3% and 15.2% of total protein-bound ceramides, respectively (Fig. 4B and supplemental Table S8). The major LCB chain lengths in the protein-bound ceramides were d/t16–22, of which d/t20 (44.2%) was the most abundant, followed by d/t18 (24.4%) (Fig. 4B and supplemental Table S9). The levels of protein-bound ceramides containing d/t23–26 were low. The percentages of LCBs in P-OS were 47.7% (d20), 21.1% (d18), and 9.6% (d22) and in P-OH were 36.3% (t18), 32.7% (t20), and 10.6% (t19) (Fig. 4C and supplemental Table S10). There was almost no difference in FA composition between the ceramide species with LCBs of different chain lengths. The predominant FA chain length was C30:0 (∼45%), followed by C32:0 and C32:1 (∼15% each), in both P-OS and P-OH (Fig. 4D and supplemental Table S11). We thus revealed that protein-bound ceramides containing d/t16–26 are present in the human SC.

### The chain-length diversity produced by different combinations of SPT subunits

**Fig. 5:**
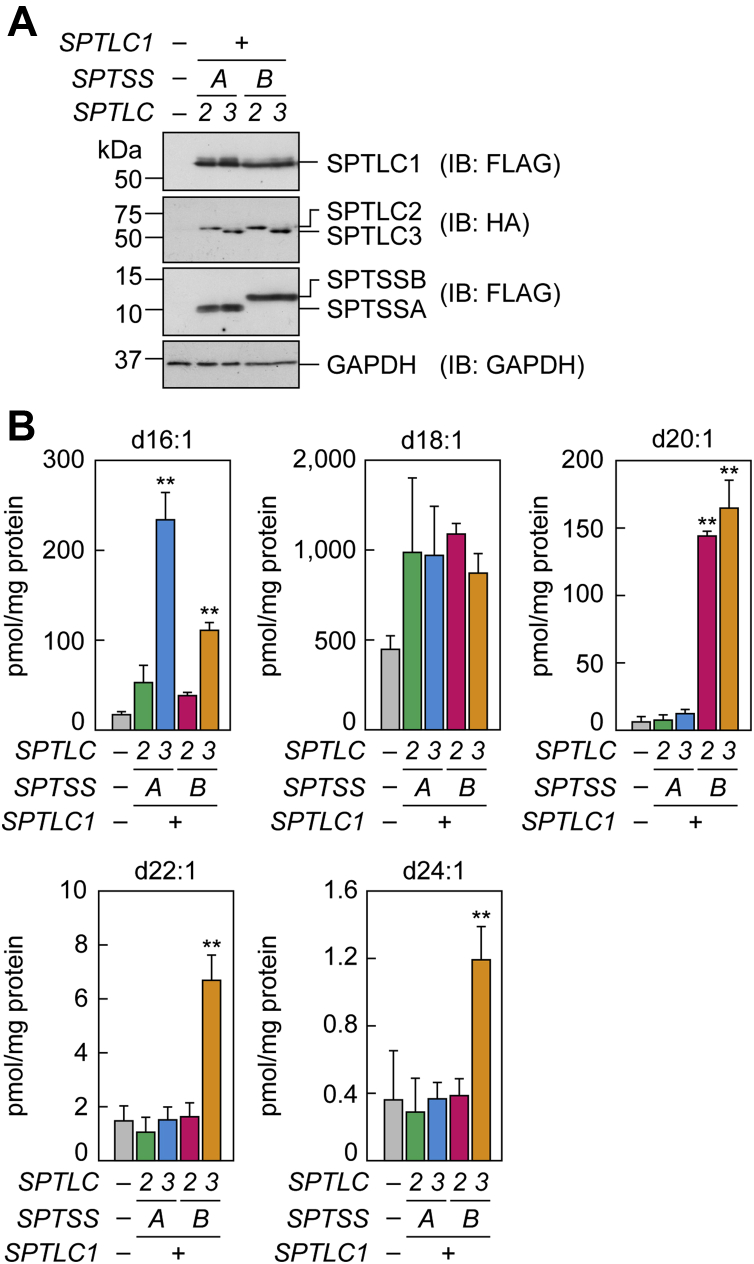
Production of ceramide species with LCBs of specific chain lengths by different combinations of SPT subunits. HEK 293T cells were transfected with pCE-puro 3×FLAG-1 (vector), pCE-puro 3×FLAG-SPTLC1, pCE-puro HA-SPTLC2, pCE-puro HA-SPTLC3, pCE-puro 3×FLAG-SPTSSA, and/or pCE-puro 3×FLAG-SPTSSB plasmids, as indicated, and cultured for 24 h. A: Total cell lysates (5 μg) prepared from the transfected cells were separated by SDS-PAGE and subjected to immunoblotting using anti-FLAG antibody, anti-HA antibody, or anti-GAPDH antibody (loading control). B: Lipids were extracted from the transfected cells and subjected to LC/MS/MS analysis to quantify NS ceramides with a d16:1, d18:1, d20:1, d22:1, or d24:1 LCB. Values are means + SDs of the sum of NS ceramide species containing C16–C24 FAs (n = 3, ∗∗*P* < 0.01; Dunnett’s test vs. vector control). IB, immunoblotting.

It is reported that the chain lengths of LCBs are determined by the substrate specificities of SPT complexes (38, 41, 42, 53, 54). However, the exact substrate specificities of SPT complexes, especially toward acyl-CoAs longer than C22, remained unclear. In the present study, HEK 293T cells were transfected with the plasmids for overexpressing each of the 3×FLAG-tagged or HA-tagged SPT subunits, and the expression of each SPT subunit was confirmed by immunoblotting (Fig. 5A). Lipids were then extracted from the cells, and the levels of NS ceramides containing LCBs of various chain lengths were quantified via LC/MS/MS. The levels of NS containing d16:1 were increased by overexpression of the SPTs that include SPTLC3 (SPTLC1/3/SSA and SPTLC1/3/SSB) relative to the vector control (Fig. 5B), which was consistent with the earlier reports (38, 53, 54). The overexpression of any combination of SPT subunits tended to increase the levels of d18:1 NS approximately 2-fold relative to the control cells, although this difference was not statistically significant. The d20:1 NS levels were increased ∼15-fold relative to the vector control by overexpression of the SPTs that contain SPTSSB (SPTLC1/2/SSB and SPTLC1/3/SSB). The levels of d22:1 NS and d24:1 NS were increased only in the cells overexpressing the SPT complex comprising SPTLC1/3/SSB, and these levels were 5-fold and 3-fold, respectively, those in the control cells. These results on the substrate specificities of SPT complexes in the generation of ≤d/t22 LCBs were generally consistent with previous reports (38, 41, 42, 53, 54). In addition to these results, we demonstrated that SPTLC1/3/SSB is involved in the production of LCBs with d/t24.

### Increased expression of SPT subunits during keratinocyte differentiation

**Fig. 6:**
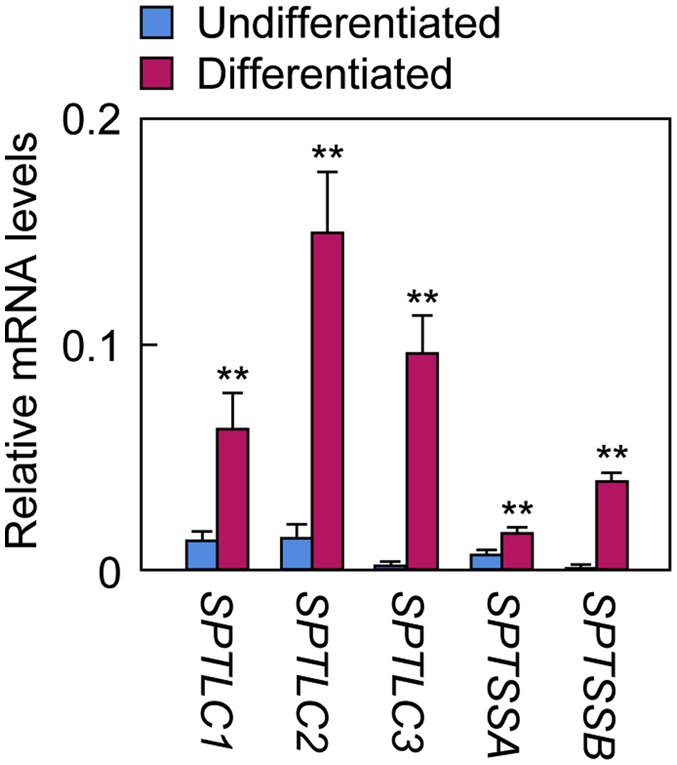
Expression of SPT subunits during human keratinocyte differentiation. Total RNA was prepared from human primary keratinocytes that were either undifferentiated or differentiated for 7 days and subjected to quantitative real-time RT-PCR using specific primers for SPT subunits (*SPTLC1*, *SPTLC2*, *SPTLC3*, *SPTSSA*, and *SPTSSB*) and the housekeeping gene *GAPDH*. Values are the means + SDs of the quantity of each mRNA relative to that of *GAPDH* (n = 3, ∗∗*P* < 0.01; Student’s *t*-test).

Our findings that ceramide species containing LCBs with a wide range of chain lengths are present in the human SC (Figs. 3, 4) suggest that various SPT subunits are highly expressed in the human epidermis, especially in the stratum granulosum, where ceramide synthesis is active. To confirm this, we prepared total RNA from human primary keratinocytes and investigated the expression levels of SPT subunits via quantitative real-time RT-PCR. *SPTLC1*, *SPTLC2*, and *SPTSSA* were expressed in undifferentiated keratinocytes, but *SPTLC3* and *SPTSSB* were barely expressed (Fig. 6). By contrast, all *SPT* subunits were highly expressed in differentiated keratinocytes. Compared with undifferentiated keratinocytes, the mRNA levels of *SPTLC1*, *SPTLC2*, and *SPTSSA* were increased 5-, 10-, and 3-fold, respectively, in differentiated keratinocytes, whereas those of *SPTLC3* and *SPTSSB* were greatly increased (174- and 2,623-fold respectively). These results indicate that the existence of ceramide species containing LCBs with various chain lengths in the human SC is attributable to the expression of all SPT subunits.

## Discussion

To date, comprehensive analyses of human SC ceramides have been performed via LC/MS or LC/MS/MS using the product ion scanning mode. LC/MS analyses by two research groups have revealed the presence of 264 or 182 ceramides (20, 50), but they only specified the ceramide species based on the sum of their LCB and FA chain lengths. LC/MS/MS analysis using the product ion scanning mode detected 342 ceramides in human SC by discriminating between LCB chain lengths (19). However, because of the low sensitivity of product ion scanning analysis, it is unlikely that all ceramide species were covered. Another product ion scanning analysis predicted the presence of >1,000 ceramide species in the human SC (20), but the actual number of ceramides has not been determined. In the present study, using LC/MS/MS in MRM mode, we identified and quantified 1,327 unbound and 254 protein-bound ceramides with d/t16–26 LCBs (Figs. 3, 4), thus elucidating the whole picture of human SC ceramides.

**Fig. 7:**
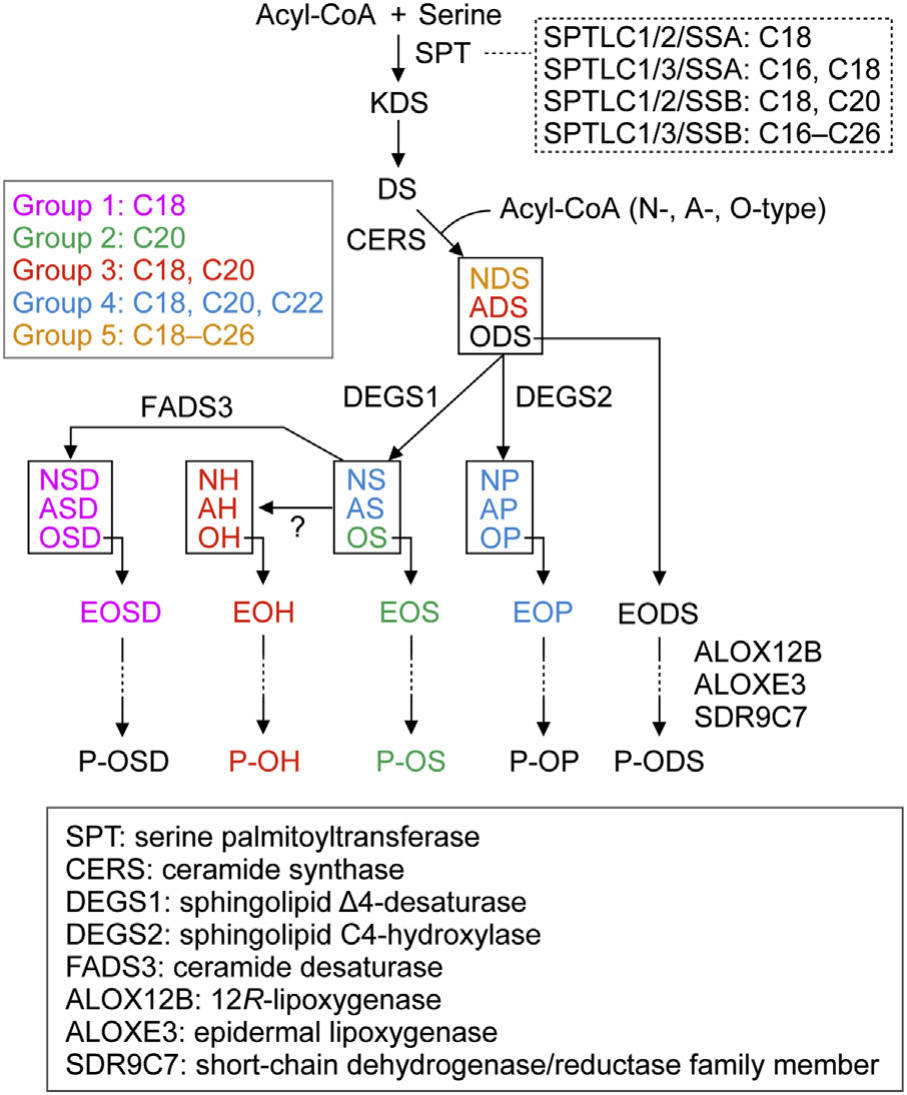
Molecular mechanism behind the generation of diverse LCB chain lengths. The biosynthetic pathway of each ceramide class and the proteins involved in each reaction step are shown. In the de novo ceramide synthesis pathway, SPT complexes catalyze the condensation of acyl-CoA with serine to produce KDS. The four SPT complexes produce KDSs with different chain lengths, as shown in the dotted box. Next, KDS is converted to DS and condensed with acyl-CoA (N-, A-, or O-type) by CERS, generating DS-type ceramides. These are converted to S- and P-type ceramides by DEGS1 and DEGS2, respectively. SD-type ceramides are produced by FADS3, which desaturates the C14 position of the LCB moiety in S-type ceramides. H-type ceramides are generated by an unidentified enzyme, which is assumed to hydroxylate the C6 position of the LCB moiety in S-type ceramides. O-type ceramides are converted to acylceramides (EO-type ceramides), and some acylceramides are metabolized to protein-bound ceramides (P-O-type ceramides) via several reactions, including ALOX12B-, ALOXE3-, and SDR9C7-mediated reactions. We divided all ceramide classes into five groups (color coded) based on their LCB chain-length distribution patterns. The enzyme names of the proteins presented in the figure are listed in the box at the bottom of the figure.

Each ceramide class had a characteristic LCB chain-length composition (Fig. 3D, E), and based on this, we classified all unbound ceramide classes into five groups (Figs. 3E, 7). It is likely that the differences in the LCB chain-length composition among these groups result from the substrate specificities of the enzymes involved in ceramide metabolism. DS-type ceramides are produced via the condensation of DS and acyl-CoA, which is catalyzed by ceramide synthases (CERS1–6). S-type ceramides and P-type ceramides are generated from DS-type ceramides by the sphingolipid Δ4-desaturase DEGS1 and the sphingolipid C4-hydroxylase DEGS2, respectively. Most S- and P-type ceramides, such as NS, AS, NP, AP, OP, and EOP, belonged to group 4 (Figs. 3E, 7). In this group, the proportions of d/t18, d/t20, and d/t22 species were high (11–33% each), whereas those of d/t24 and d/t26 species were low (Fig. 3E). We speculate that these LCB chain-length distributions resulted from low levels of activity of DEGS1 and DEGS2 toward DS-type ceramides composed with d24–26 LCBs.

Group 3 ceramides were mostly H-type ceramides, among which t18 and t20 species were predominant (Figs. 3E, 7). Although the production pathway of H-type ceramides has not been revealed, they are thought to be produced by the C6-hydroxylation of S-type ceramides by an unidentified hydroxylase. This unidentified enzyme, therefore, may exhibit a high substrate specificity toward S-type ceramides with d18 or d20.

Group 1 ceramides included only SD-type ceramides, of which more than 90% were d18 species and a trace quantity was d20 species (Figs. 3E, 7). The ceramide desaturase FADS3 introduces a *cis* double bond between C14 and C15 of the LCB moiety in S-type ceramides to produce SD-type ceramides (55). The extremely high proportion of d18 species in SD-type ceramides therefore suggests that FADS3 exhibits a high substrate specificity toward S-type ceramides with d18.

Group 2 ceramides included OS and EOS (Figs. 3E, 7). In this group, d20 species were particularly abundant (about 40%) (Fig. 3E), and this may be attributable to the substrate specificity of CERS3. Mammalian CERSs (CERS1–6) have different substrate specificities toward acyl-CoAs, and CERS3 shows high activity toward acyl-CoAs with a chain length of C26 or more (25, 29). CERS3 catalyzes the condensation of DS and O-type acyl-CoAs with C30–36 to generate ODS, the precursor of OS. Although the LCB chain-length composition of ODS is unclear because of the extremely low abundance of ODS, we speculate that CERS3 may exhibit high activity toward d20.

Group 5 ceramides included only NDS (Figs. 3E, 7). The NDS contained a wide range of LCB chain lengths (d16–26) and was characterized by abundant d24 and d26 species (Fig. 3E). This ceramide class is the remnant that is not converted to NS and NP by DEGS1 and DEGS2, respectively. We therefore speculate that d24 and d26 NDS species remain unmetabolized because of the low levels of activity of DEGS1 and DEGS2 toward them, as described above.

The levels of ceramides with LCBs longer than d/t22 were low in both protein-bound ceramides (P-O-type ceramides) and acylceramides (EO-type ceramides), and the LCB chain-length composition of protein-bound ceramides was similar to that of acylceramides (Figs. 3, 4). Protein-bound ceramides are generated from acylceramides (11, 15, 17, 56), which suggests that the enzymes involved in the conversion of acylceramides to protein-bound ceramides do not exhibit strict substrate specificities with respect to LCB chain lengths. During the conversion of acylceramides to protein-bound ceramides, the linoleic acid moiety undergoes peroxidation, epoxidation plus hydroxylation, and oxidation, and these reactions are catalyzed by the 12*R*-lipoxygenase ALOX12B, epidermal lipoxygenase-3 ALOXE3, and short-chain dehydrogenase/reductase family member SDR9C7, respectively (Fig. 7) (11, 27, 57). Mutations in any of these genes cause congenital ichthyosis in humans and neonatal lethality (because of skin barrier abnormalities) in mice (11, 26, 27, 58), indicating the importance of protein-bound ceramides in skin barrier formation. Protein-bound ceramides in the corneocyte cell surface are thought to function as scaffolds for the lipid lamellae (15, 17). In addition, they may provide resistance against detergents and chemical stability to corneocytes by covalently binding to proteins.

It has been reported that the molecular mechanism behind the chain-length diversity of LCBs is the differences in substrate specificity among four types of SPT complex (38, 41, 42, 53, 54). In the present study, we expanded our knowledge of the molecular mechanism that generates the diversity of LCBs. Until now, the SPT complexes involved in the production of d/t24 and longer LCBs had not yet been identified, but in this study, we were able to show that the SPTLC1/3/SSB complex is responsible for the production of d/t24 LCBs (Fig. 5). The SPTLC1/3/SSB complex may also be involved in the production of LCBs with d/t26, although we could not prove this using our assay system. The expression of *SPTLC3* and *SPTSSB* was strongly induced by the differentiation of human keratinocytes (Fig. 6). Expression of *SPTSSB* in differentiated keratinocytes is dependent on the transcription factor forkhead box C1 (FOXC1) (59). We also revealed that all SPT subunits were highly expressed in the differentiated keratinocytes (Fig. 6). These findings indicate that the chain-length diversity of LCBs in the human SC results from the existence of these four types of SPT complex.

The skin barrier function is extremely important for protection against infection and prevention of loss of water and electrolytes. In patients with atopic dermatitis and psoriasis, the total chain length (the sum of FA and LCB chain lengths) of ceramides is reduced (8, 9, 60). Intermolecular interactions among ceramides are reported to be affected more by the chain length of the LCB moiety than that of the FA moiety (61). Therefore, we predict that the presence of ceramides with longer LCBs is required for appropriate lipid lamellae formation and normal skin barrier function.

In this study, we have revealed the detailed chain-length distribution of the LCBs in each ceramide class in the human SC. This study is the first to uncover the whole picture of the diversity of human SC ceramides. Although it has been reported that ceramide composition is altered in patients with atopic dermatitis, psoriasis, and ichthyosis (8, 24, 25, 62, 63), comprehensive measurement of ceramides, including LCB chain-length diversity, as performed in this study, has not been done before. In the future, comprehensive ceramide measurement in patients with these skin diseases will be required to elucidate the relationship between changes in ceramide composition and the pathology of each disease, as well as to develop methods of diagnosis and treatment for these diseases.

## Data availability

All data are included in the article.

## Supplemental data

This article contains supplemental data.

## Conflict of interest

The authors declare that they have no conflicts of interest with the contents of this article.
