# Supplementary material for: Whole picture of human stratum corneum ceramides, including the chain-length diversity of long-chain bases
Source: J Lipid Res. 2022 May 30;63(7):100235. doi: 10.1016/j.jlr.2022.100235 (PMC9240646; doi:10.1016/j.jlr.2022.100235)
Supplement: Supplemental Table S1 [file mmc1.docx]

**Supplemental Table S1.** Primers used for DNA cloning in this study

| Primer name | Sequence |
| --- | --- |
| SPTLC1-F | 5'-GGATCCATGGCGACCGCCACGGAGCAGTGGG-3' |
| SPTLC1-R | 5'-CTAGAGCAGGACGGCCTGGGCTACC-3' |
| SPTLC2-F | 5'-AGATCTATGCGGCCGGAGCCCGGAGGCTGCTG-3' |
| SPTLC2-R | 5'-TCAGTCTTCTGTTTCTTCATACGTC-3' |
| SPTLC3-F | 5'-GGATCCATGGCTAACCCTGGAGGTGGTGCTG-3' |
| SPTLC3-R | 5'-TTAATCTTCGAGTTCAAAGCTCGTC-3' |
| SPTSSA-F | 5'-GGATCCATGGCGGGGATGGCGCTGGCGCGGG-3' |
| SPTSSA-R | 5'-TCATTGTACGATTTCAAAGTAGTGC-3' |
| SPTSSB-F | 5'-GGATCCATGGATTTGAGGCGTGTGAAGG-3' |
| SPTSSB-R | 5'-TCAATTAGAAATTGTACTGTGATATC-3' |
