# Supplementary material for: Whole picture of human stratum corneum ceramides, including the chain-length diversity of long-chain bases
Source: J Lipid Res. 2022 May 30;63(7):100235. doi: 10.1016/j.jlr.2022.100235 (PMC9240646; doi:10.1016/j.jlr.2022.100235)
Supplement: Supplemental Table S3 [file mmc3.docx]

**Supplemental Table S3.** Primers used for quantitative real-time RT-PCR in this study

| Primer name | Sequence |
| --- | --- |
| SPTLC1-F2 | 5'-GAAGAGAGCACTGGGTCTCG-3' |
| SPTLC1-R2 | 5'-TGTTCCACCGTGACCACAAC-3' |
| SPTLC2-F2 | 5'-TGGGTTCCTACAACTATCTTGGA-3' |
| SPTLC2-R2 | 5'-CATACGCCATAGCAGCTTCTAC -3' |
| SPTLC3-F2 | 5'-GTTTTAGAGGTGTATGGCACAGG-3' |
| SPTLC3-R2 | 5'-TCAGGAACTTAGCCACAAGGT-3' |
| SPTSSA-F2 | 5'-CTGGTCACGGCGCTCTAC-3' |
| SPTSSA-R2 | 5'-ATGTGCTGGGGCATGAAG-3' |
| SPTSSB-F2 | 5'-TTTTAGAGCCCTGGGAGCGA-3' |
| SPTSSB-R2 | 5'-ATTCCCAAGCCAGGCGAATG-3' |
| GAPDH-F | 5'-GAACGGGAAGCTCACTGGCATGGCC-3' |
| GAPDH-R | 5'-TGTCATACCAGGAAATGAGCTTGAC-3' |
