# Supplementary material for: Whole picture of human stratum corneum ceramides, including the chain-length diversity of long-chain bases
Source: J Lipid Res. 2022 May 30;63(7):100235. doi: 10.1016/j.jlr.2022.100235 (PMC9240646; doi:10.1016/j.jlr.2022.100235)
Supplement: Supplemental Table S4 [file mmc4.docx]

**Supplemental Table S4.** Quantities and proportions of unbound ceramide classes

| Ceramide class | pmol/mg protein | % |
| --- | --- | --- |
| NS | 859.2 ± 381.3 | 3.4 ± 0.4 |
| NDS | 2864.7 ± 1098.6 | 11.3 ± 1.3 |
| NH | 5908.2 ± 2822.3 | 23.4 ± 3.2 |
| NP | 7437.4 ± 2841.0 | 29.4 ± 3.3 |
| NSD | 1.3 ± 0.7 | 0.005 ± 0.004 |
| AS | 1007.8 ± 361.4 | 4.0 ± 1.0 |
| ADS | 228.6 ± 153.8 | 0.9 ± 0.7 |
| AH | 2303.0 ± 925.3 | 9.1 ± 1.8 |
| AP | 1621.7 ± 946.7 | 6.4 ± 2.6 |
| ASD | 6.1 ± 3.0 | 0.02 ± 0.01 |
| OS | 227.4 ± 108.2 | 0.9 ± 0.2 |
| ODS | n.d. | n.d. |
| OH | 48.8 ± 18.6 | 0.2 ± 0.03 |
| OP | 44.1 ± 17.1 | 0.2 ± 0.1 |
| OSD | 0.4 ± 0.2 | 0.002 ± 0.002 |
| EOS | 1955.0 ± 869.2 | 7.7 ± 1.2 |
| EODS | n.d. | n.d. |
| EOH | 508.9 ± 241.8 | 2.0 ± 0.3 |
| EOP | 265.9 ± 138.0 | 1.1 ± 0.4 |
| EOSD | 0.5 ± 0.3 | 0.002 ± 0.001 |
| Total | 25288.8 ± 9869.2 |  |

n.d., not detected.
