# Supplementary material for: Whole picture of human stratum corneum ceramides, including the chain-length diversity of long-chain bases
Source: J Lipid Res. 2022 May 30;63(7):100235. doi: 10.1016/j.jlr.2022.100235 (PMC9240646; doi:10.1016/j.jlr.2022.100235)
Supplement: Supplemental Table S5 [file mmc5.docx]

**Supplemental Table S5.** Total quantities and proportions of unbound ceramides with each LCB chain-length category

| LCB | pmol/mg protein | % |
| --- | --- | --- |
| d/t16 | 1551.9 ± 650.3 | 6.1 ± 2.6 |
| d/t17 | 2245.8 ± 955.3 | 8.9 ± 3.8 |
| d/t18 | 7225.2 ± 3026.9 | 28.6 ± 12 |
| d/t19 | 1130.2 ± 462.9 | 4.5 ± 1.8 |
| d/t20 | 6277.4 ± 2497.1 | 24.8 ± 9.9 |
| d/t21 | 1232.2 ± 464.1 | 4.9 ± 1.8 |
| d/t22 | 3242.7 ± 1217.3 | 12.8 ± 4.8 |
| d/t23 | 262.8 ± 89.8 | 1.0 ± 0.4 |
| d/t24 | 1064.5 ± 389.6 | 4.2 ± 1.5 |
| d/t25 | 220.8 ± 82.7 | 0.9 ± 0.3 |
| d/t26 | 835.4 ± 283.2 | 3.3 ± 1.1 |
| Total | 25288.8 ± 9869.2 |  |
