# Supplementary material for: Whole picture of human stratum corneum ceramides, including the chain-length diversity of long-chain bases
Source: J Lipid Res. 2022 May 30;63(7):100235. doi: 10.1016/j.jlr.2022.100235 (PMC9240646; doi:10.1016/j.jlr.2022.100235)
Supplement: Supplemental Table S6 [file mmc6.docx]

**Supplemental Table S6.** The total quantity of ceramides with each LCB chain length in each ceramide class

| Ceramide class | LCB | pmol/mg protein | % (in each class) |
| --- | --- | --- | --- |
|  |  |  |  |
| NS | d16:1 | 80.0 ± 33.7 | 9.7 ± 2.4 |
| NS | d17:1 | 54.5 ± 26.8 | 6.3 ± 0.6 |
| NS | d18:1 | 172.9 ± 83.0 | 20.0 ± 0.8 |
| NS | d19:1 | 35.7 ± 19.3 | 4.0 ± 0.4 |
| NS | d20:1 | 226.8 ± 102.9 | 26.4 ± 1.8 |
| NS | d21:1 | 48.1 ± 21.8 | 5.6 ± 0.5 |
| NS | d22:1 | 153.6 ± 66.7 | 17.8 ± 1.7 |
| NS | d23:1 | 10.2 ± 4.5 | 1.2 ± 0.2 |
| NS | d24:1 | 33.5 ± 15 | 3.9 ± 0.5 |
| NS | d25:1 | 9.9 ± 4.5 | 1.2 ± 0.3 |
| NS | d26:1 | 33.8 ± 12.4 | 4.0 ± 0.6 |
| NDS | d16:0 | 119.7 ± 46.9 | 4.3 ± 0.7 |
| NDS | d17:0 | 56.7 ± 28.8 | 1.9 ± 0.4 |
| NDS | d18:0 | 322.5 ± 163.1 | 11.0 ± 2.0 |
| NDS | d19:0 | 67.5 ± 27.4 | 2.4 ± 0.3 |
| NDS | d20:0 | 362.3 ± 132.7 | 12.9 ± 2.8 |
| NDS | d21:0 | 96.2 ± 36.4 | 3.4 ± 0.5 |
| NDS | d22:0 | 354.4 ± 125.8 | 12.5 ± 1.1 |
| NDS | d23:0 | 83.2 ± 33.7 | 2.9 ± 0.5 |
| NDS | d24:0 | 702.0 ± 274 | 24.5 ± 1.4 |
| NDS | d25:0 | 148.6 ± 63.9 | 5.1 ± 0.6 |
| NDS | d26:0 | 551.5 ± 220.0 | 19.2 ± 1.3 |
| NH | t16:1 | 212.6 ± 98.1 | 3.7 ± 0.6 |
| NH | t17:1 | 513.4 ± 234.6 | 8.8 ± 0.7 |
| NH | t18:1 | 2439.2 ± 1081.2 | 41.7 ± 1.1 |
| NH | t19:1 | 405.7 ± 208.3 | 6.8 ± 0.4 |
| NH | t20:1 | 1923.2 ± 1009.5 | 32.2 ± 1.3 |
| NH | t21:1 | 191.1 ± 97.4 | 3.2 ± 0.4 |
| NH | t22:1 | 223.1 ± 112 | 3.7 ± 0.4 |
| NH | t23:1 | n.d. | n.d. |
| NH | t24:1 | n.d. | n.d. |
| NH | t25:1 | n.d. | n.d. |
| NH | t26:1 | n.d. | n.d. |
| NP | t16:0 | 601.4 ± 285.3 | 8.1 ± 1.7 |
| NP | t17:0 | 852.4 ± 411.4 | 11.3 ± 1.4 |
| NP | t18:0 | 2007.8 ± 926.6 | 26.6 ± 2.4 |
| NP | t19:0 | 208.0 ± 81.0 | 2.8 ± 0.3 |
| NP | t20:0 | 1190.7 ± 377.5 | 16.4 ± 2.1 |
| NP | t21:0 | 467.6 ± 175.5 | 6.3 ± 0.8 |
| NP | t22:0 | 1453.3 ± 546.6 | 19.5 ± 2 |
| NP | t23:0 | 128.4 ± 40.8 | 1.8 ± 0.4 |
| NP | t24:0 | 260.0 ± 86.0 | 3.6 ± 0.6 |
| NP | t25:0 | 52.3 ± 13.7 | 0.8 ± 0.2 |
| NP | t26:0 | 215.6 ± 64.1 | 3.1 ± 0.9 |
| NSD | d16:2 | n.d. | n.d. |
| NSD | d17:2 | n.d. | n.d. |
| NSD | d18:2 | 1.1 ± 0.7 | 97.8 ± 1.6 |
| NSD | d19:2 | n.d. | n.d. |
| NSD | d20:2 | 0.03 ± 0.02 | 2.2 ± 1.6 |
| NSD | d21:2 | n.d. | n.d. |
| NSD | d22:2 | n.d. | n.d. |
| NSD | d23:2 | n.d. | n.d. |
| NSD | d24:2 | n.d. | n.d. |
| NSD | d25:2 | n.d. | n.d. |
| NSD | d26:2 | n.d. | n.d. |
| AS | d16:1 | 140.8 ± 52.7 | 14.2 ± 3 |
| AS | d17:1 | 91.0 ± 33.1 | 9.1 ± 0.9 |
| AS | d18:1 | 295.5 ± 111.5 | 29.3 ± 2.5 |
| AS | d19:1 | 50.9 ± 18.4 | 5.1 ± 0.5 |
| AS | d20:1 | 239.1 ± 87.3 | 23.7 ± 1.9 |
| AS | d21:1 | 44.9 ± 20.3 | 4.3 ± 0.9 |
| AS | d22:1 | 108.3 ± 43.6 | 10.6 ± 1.8 |
| AS | d23:1 | 6.9 ± 3.2 | 0.7 ± 0.2 |
| AS | d24:1 | 15.9 ± 6.2 | 1.6 ± 0.3 |
| AS | d25:1 | 3.3 ± 1.2 | 0.3 ± 0.1 |
| AS | d26:1 | 11.4 ± 4.8 | 1.1 ± 0.3 |
| ADS | d16:0 | 5.8 ± 5.0 | 2.8 ± 1.4 |
| ADS | d17:0 | 12.8 ± 11.1 | 5.7 ± 1.6 |
| ADS | d18:0 | 79.6 ± 58.7 | 35.2 ± 5.8 |
| ADS | d19:0 | 11.7 ± 8.4 | 5.3 ± 1.5 |
| ADS | d20:0 | 90.8 ± 62.0 | 38.0 ± 6.2 |
| ADS | d21:0 | 9.1 ± 5.3 | 4.8 ± 2.3 |
| ADS | d22:0 | 18.8 ± 12.6 | 8.1 ± 2.5 |
| ADS | d23:0 | n.d. | n.d. |
| ADS | d24:0 | n.d. | n.d. |
| ADS | d25:0 | n.d. | n.d. |
| ADS | d26:0 | n.d. | n.d. |
| AH | t16:1 | 140.4 ± 54.5 | 6.3 ± 1.4 |
| AH | t17:1 | 276.8 ± 107.7 | 12.1 ± 0.8 |
| AH | t18:1 | 938.1 ± 383.4 | 40.8 ± 1.9 |
| AH | t19:1 | 156.2 ± 61.9 | 6.8 ± 0.8 |
| AH | t20:1 | 692.9 ± 297.0 | 29.8 ± 2.5 |
| AH | t21:1 | 49.8 ± 20.4 | 2.1 ± 0.5 |
| AH | t22:1 | 48.8 ± 23.4 | 2.1 ± 0.4 |
| AH | t23:1 | n.d. | n.d. |
| AH | t24:1 | n.d. | n.d. |
| AH | t25:1 | n.d. | n.d. |
| AH | t26:1 | n.d. | n.d. |
| AP | t16:0 | 207.5 ± 124.3 | 13.6 ± 4.3 |
| AP | t17:0 | 271 ± 140.8 | 17.8 ± 3.4 |
| AP | t18:0 | 421.5 ± 281.3 | 24.9 ± 3.5 |
| AP | t19:0 | 44.9 ± 38.1 | 2.6 ± 1.0 |
| AP | t20:0 | 302.5 ± 179.2 | 18.5 ± 2.4 |
| AP | t21:0 | 85.2 ± 44.9 | 5.5 ± 1.5 |
| AP | t22:0 | 266.4 ± 168.6 | 15.8 ± 3.8 |
| AP | t23:0 | 12.9 ± 9 | 0.8 ± 0.4 |
| AP | t24:0 | 8.9 ± 12.2 | 0.5 ± 0.7 |
| AP | t25:0 | 0.3 ± 0.7 | 0.01 ± 0.03 |
| AP | t26:0 | 0.8 ± 1.3 | 0.04 ± 0.05 |
| ASD | d16:2 | 0.004 ± 0.008 | 0.1 ± 0.2 |
| ASD | d17:2 | n.d. | n.d. |
| ASD | d18:2 | 5.9 ± 3.0 | 96.7 ± 1.3 |
| ASD | d19:2 | n.d. | n.d. |
| ASD | d20:2 | 0.2 ± 0.1 | 3.2 ± 1.4 |
| ASD | d21:2 | n.d. | n.d. |
| ASD | d22:2 | n.d. | n.d. |
| ASD | d23:2 | n.d. | n.d. |
| ASD | d24:2 | n.d. | n.d. |
| ASD | d25:2 | n.d. | n.d. |
| ASD | d26:2 | n.d. | n.d. |
| OS | d16:1 | 5.4 ± 2.6 | 2.5 ± 0.5 |
| OS | d17:1 | 9.2 ± 4.2 | 4.1 ± 0.6 |
| OS | d18:1 | 38.7 ± 18.5 | 17.1 ± 1.5 |
| OS | d19:1 | 12.9 ± 6.2 | 5.7 ± 0.6 |
| OS | d20:1 | 96.8 ± 50.2 | 42.2 ± 2.5 |
| OS | d21:1 | 18.6 ± 8.3 | 8.2 ± 1.0 |
| OS | d22:1 | 40.6 ± 18.3 | 17.9 ± 2.3 |
| OS | d23:1 | 1.3 ± 0.7 | 0.6 ± 0.1 |
| OS | d24:1 | 2.0 ± 1.0 | 0.9 ± 0.1 |
| OS | d25:1 | 0.3 ± 0.1 | 0.1 ± 0.1 |
| OS | d26:1 | 1.6 ± 0.9 | 0.7 ± 0.4 |
| ODS | d16:0 | n.d. | n.d. |
| ODS | d17:0 | n.d. | n.d. |
| ODS | d18:0 | n.d. | n.d. |
| ODS | d19:0 | n.d. | n.d. |
| ODS | d20:0 | n.d. | n.d. |
| ODS | d21:0 | n.d. | n.d. |
| ODS | d22:0 | n.d. | n.d. |
| ODS | d23:0 | n.d. | n.d. |
| ODS | d24:0 | n.d. | n.d. |
| ODS | d25:0 | n.d. | n.d. |
| ODS | d26:0 | n.d. | n.d. |
| OH | t16:1 | n.d. | n.d. |
| OH | t17:1 | 2.9 ± 1.1 | 6.4 ± 2.4 |
| OH | t18:1 | 17.8 ± 6.1 | 37.1 ± 3.3 |
| OH | t19:1 | 2.5 ± 1.2 | 5.2 ± 2.0 |
| OH | t20:1 | 22.3 ± 10.5 | 44.6 ± 5.3 |
| OH | t21:1 | 1.6 ± 1.1 | 3.2 ± 2.1 |
| OH | t22:1 | 1.7 ± 0.6 | 3.6 ± 1.0 |
| OH | t23:1 | n.d. | n.d. |
| OH | t24:1 | n.d. | n.d. |
| OH | t25:1 | n.d. | n.d. |
| OH | t26:1 | n.d. | n.d. |
| OP | t16:0 | 2.6 ± 1.6 | 6.0 ± 3.0 |
| OP | t17:0 | 9.9 ± 7.2 | 21.4 ± 8.5 |
| OP | t18:0 | 14.3 ± 6.5 | 33.1 ± 9.7 |
| OP | t19:0 | n.d. | n.d. |
| OP | t20:0 | 9.1 ± 5.2 | 20.3 ± 9.2 |
| OP | t21:0 | n.d. | n.d. |
| OP | t22:0 | 8.2 ± 4.9 | 19.3 ± 10.5 |
| OP | t23:0 | n.d. | n.d. |
| OP | t24:0 | n.d. | n.d. |
| OP | t25:0 | n.d. | n.d. |
| OP | t26:0 | n.d. | n.d. |
| OSD | d16:2 | n.d. | n.d. |
| OSD | d17:2 | n.d. | n.d. |
| OSD | d18:2 | 0.4 ± 0.2 | 100 ± 0 |
| OSD | d19:2 | n.d. | n.d. |
| OSD | d20:2 | n.d. | n.d. |
| OSD | d21:2 | n.d. | n.d. |
| OSD | d22:2 | n.d. | n.d. |
| OSD | d23:2 | n.d. | n.d. |
| OSD | d24:2 | n.d. | n.d. |
| OSD | d25:2 | n.d. | n.d. |
| OSD | d26:2 | n.d. | n.d. |
| EOS | d16:1 | 16.6 ± 8.4 | 0.9 ± 0.3 |
| EOS | d17:1 | 47.9 ± 22.8 | 2.5 ± 0.6 |
| EOS | d18:1 | 233.0 ± 106.2 | 12 ± 1.6 |
| EOS | d19:1 | 92.0 ± 46.2 | 4.6 ± 0.5 |
| EOS | d20:1 | 835.7 ± 386.5 | 42.7 ± 2.0 |
| EOS | d21:1 | 178.6 ± 77.6 | 9.1 ± 1.0 |
| EOS | d22:1 | 479.1 ± 208.4 | 24.5 ± 2.6 |
| EOS | d23:1 | 15.7 ± 6.8 | 0.8 ± 0.2 |
| EOS | d24:1 | 34.3 ± 13.8 | 1.8 ± 0.2 |
| EOS | d25:1 | 5.1 ± 1.9 | 0.3 ± 0.1 |
| EOS | d26:1 | 17.1 ± 6.7 | 0.9 ± 0.2 |
| EODS | d16:0 | n.d. | n.d. |
| EODS | d17:0 | n.d. | n.d. |
| EODS | d18:0 | n.d. | n.d. |
| EODS | d19:0 | n.d. | n.d. |
| EODS | d20:0 | n.d. | n.d. |
| EODS | d21:0 | n.d. | n.d. |
| EODS | d22:0 | n.d. | n.d. |
| EODS | d23:0 | n.d. | n.d. |
| EODS | d24:0 | n.d. | n.d. |
| EODS | d25:0 | n.d. | n.d. |
| EODS | d26:0 | n.d. | n.d. |
| EOH | t16:1 | 13.0 ± 6.9 | 2.5 ± 0.3 |
| EOH | t17:1 | 26.8 ± 12.8 | 5.3 ± 0.9 |
| EOH | t18:1 | 169.9 ± 68.3 | 34.3 ± 3.1 |
| EOH | t19:1 | 32.8 ± 16.5 | 6.4 ± 0.6 |
| EOH | t20:1 | 225.2 ± 120.2 | 43.5 ± 2.8 |
| EOH | t21:1 | 22.9 ± 11.3 | 4.5 ± 0.3 |
| EOH | t22:1 | 18.1 ± 9.4 | 3.6 ± 0.8 |
| EOH | t23:1 | 0.2 ± 0.2 | 0.03 ± 0.03 |
| EOH | t24:1 | n.d. | n.d. |
| EOH | t25:1 | n.d. | n.d. |
| EOH | t26:1 | n.d. | n.d. |
| EOP | t16:0 | 6.2 ± 4.7 | 2.4 ± 0.9 |
| EOP | t17:0 | 20.7 ± 13.8 | 7.5 ± 1.9 |
| EOP | t18:0 | 66.4 ± 44.5 | 24.2 ± 4.2 |
| EOP | t19:0 | 9.4 ± 5.4 | 3.5 ± 1.4 |
| EOP | t20:0 | 59.7 ± 30.5 | 22.5 ± 3.3 |
| EOP | t21:0 | 18.6 ± 9.3 | 7.2 ± 1.5 |
| EOP | t22:0 | 68.4 ± 30.9 | 26.3 ± 3.1 |
| EOP | t23:0 | 4.1 ± 2.1 | 1.6 ± 0.5 |
| EOP | t24:0 | 7.9 ± 3.9 | 3.2 ± 1.0 |
| EOP | t25:0 | 1.0 ± 1.0 | 0.3 ± 0.3 |
| EOP | t26:0 | 3.6 ± 2.2 | 1.4 ± 0.3 |
| EOSD | d16:2 | n.d. | n.d. |
| EOSD | d17:2 | n.d. | n.d. |
| EOSD | d18:2 | 0.5 ± 0.3 | 92.5 ± 6.8 |
| EOSD | d19:2 | n.d. | n.d. |
| EOSD | d20:2 | 0.03 ± 0.03 | 7.5 ± 6.8 |
| EOSD | d21:2 | n.d. | n.d. |
| EOSD | d22:2 | n.d. | n.d. |
| EOSD | d23:2 | n.d. | n.d. |
| EOSD | d24:2 | n.d. | n.d. |
| EOSD | d25:2 | n.d. | n.d. |
| EOSD | d26:2 | n.d. | n.d. |

n.d., not detected.
