# Supplementary material for: Whole picture of human stratum corneum ceramides, including the chain-length diversity of long-chain bases
Source: J Lipid Res. 2022 May 30;63(7):100235. doi: 10.1016/j.jlr.2022.100235 (PMC9240646; doi:10.1016/j.jlr.2022.100235)
Supplement: Supplemental Table S8 [file mmc8.docx]

**Supplemental Table S8.** Total quantities and proportions of protein-bound ceramide classes

| Ceramide class | pmol/mg protein | % |
| --- | --- | --- |
| P-OS | 3266.7 ± 1315.9 | 82.3 ± 2.8 |
| P-OH | 604.8 ± 252.6 | 15.2 ± 2.6 |
| P-OP | 74.6 ± 60.9 | 1.9 ± 1.3 |
| P-OSD | 19.9 ± 10.5 | 0.5 ± 0.1 |
| P-ODS | 2.3 ± 1.3 | 0.06 ± 0.04 |
| Total | 3968.3 ± 1586.0 |  |

n.d., not detected.
