# Supplementary material for: Whole picture of human stratum corneum ceramides, including the chain-length diversity of long-chain bases
Source: J Lipid Res. 2022 May 30;63(7):100235. doi: 10.1016/j.jlr.2022.100235 (PMC9240646; doi:10.1016/j.jlr.2022.100235)
Supplement: Supplemental Table S9 [file mmc9.docx]

**Supplemental Table S9.** Total quantities and proportions of protein-bound ceramides with each LCB chain-length category

| LCB | pmol/mg protein | % |
| --- | --- | --- |
| d/t16 | 117.7 ± 45.5 | 3.0 ± 1.1 |
| d/t17 | 226.6 ± 92.2 | 5.7 ± 2.3 |
| d/t18 | 968.3 ± 406.1 | 24.4 ± 10.2 |
| d/t19 | 305.4 ± 129.7 | 7.7 ± 3.3 |
| d/t20 | 1755.3 ± 703.1 | 44.2 ± 17.7 |
| d/t21 | 230.7 ± 93.3 | 5.8 ± 2.3 |
| d/t22 | 346.1 ± 139.1 | 8.7 ± 3.5 |
| d/t23 | 6.0 ± 3.0 | 0.2 ± 0.1 |
| d/t24 | 9.0 ± 4.0 | 0.2 ± 0.1 |
| d/t25 | 0.9 ± 0.7 | 0.02 ± 0.02 |
| d/t26 | 2.3 ± 1.1 | 0.06 ± 00.3 |
| Total | 3968.3 ± 1586.0 |  |

n.d., not detected.
