# Supplementary material for: Whole picture of human stratum corneum ceramides, including the chain-length diversity of long-chain bases
Source: J Lipid Res. 2022 May 30;63(7):100235. doi: 10.1016/j.jlr.2022.100235 (PMC9240646; doi:10.1016/j.jlr.2022.100235)
Supplement: Supplemental Table S10 [file mmc10.docx]

**Supplemental Table S10.** The total quantity of ceramides with each LCB chain length in each protein-bound ceramide class

| Ceramide class | LCB | pmol/mg protein | % (in each class) |
| --- | --- | --- | --- |
| P-OS | d16:1 | 76.10 ± 30.13 | 2.48 ± 0.70 |
| P-OS | d17:1 | 161.24 ± 67.72 | 5.00 ± 0.58 |
| P-OS | d18:1 | 685.90 ± 296.71 | 21.06 ± 2.17 |
| P-OS | d19:1 | 236.75 ± 102.9 | 7.18 ± 0.46 |
| P-OS | d20:1 | 1545.03 ± 627.14 | 47.66 ± 1.62 |
| P-OS | d21:1 | 214.66 ± 87.90 | 6.56 ± 0.88 |
| P-OS | d22:1 | 316.22 ± 128.28 | 9.57 ± 1.29 |
| P-OS | d23:1 | 5.56 ± 2.67 | 0.16 ± 0.05 |
| P-OS | d24:1 | 7.47 ± 3.16 | 0.22 ± 0.05 |
| P-OS | d25:1 | 0.92 ± 0.66 | 0.03 ± 0.02 |
| P-OS | d26:1 | 2.27 ± 1.09 | 0.07 ± 0.02 |
| P-ODS | d16:0 | 0.01 ± 0.02 | 0.62 ± 0.95 |
| P-ODS | d17:0 | n.d. | n.d. |
| P-ODS | d18:0 | 0.53 ± 0.40 | 35.94 ± 27.25 |
| P-ODS | d19:0 | 0.09 ± 0.12 | 3.01 ± 3.47 |
| P-ODS | d20:0 | 0.51 ± 0.41 | 24.87 ± 14.84 |
| P-ODS | d21:0 | n.d. | n.d. |
| P-ODS | d22:0 | 0.22 ± 0.27 | 9.44 ± 8.15 |
| P-ODS | d23:0 | n.d. | n.d. |
| P-ODS | d24:0 | 0.51 ± 0.35 | 26.11 ± 16.07 |
| P-ODS | d25:0 | n.d. | n.d. |
| P-ODS | d26:0 | n.d. | n.d. |
| P-OH | t16:1 | 37.94 ± 16.84 | 6.33 ± 1.02 |
| P-OH | t17:1 | 51.25 ± 21.06 | 8.63 ± 0.72 |
| P-OH | t18:1 | 224.94 ± 99.11 | 36.33 ± 3.59 |
| P-OH | t19:1 | 64.27 ± 28.37 | 10.60 ± 1.08 |
| P-OH | t20:1 | 194.78 ± 82.18 | 32.72 ± 2.91 |
| P-OH | t21:1 | 13.04 ± 5.10 | 2.26 ± 0.40 |
| P-OH | t22:1 | 17.60 ± 7.50 | 2.98 ± 0.57 |
| P-OH | t23:1 | n.d. | n.d. |
| P-OH | t24:1 | 1.02 ± 1.05 | 0.15 ± 0.14 |
| P-OH | t25:1 | n.d. | n.d. |
| P-OH | t26:1 | n.d. | n.d. |
| P-OP | t16:0 | 3.34 ± 3.23 | 5.00 ± 5.77 |
| P-OP | t17:0 | 13.24 ± 13.65 | 16.11 ± 5.30 |
| P-OP | t18:0 | 36.11 ± 33.31 | 47.41 ± 5.85 |
| P-OP | t19:0 | 3.28 ± 4.41 | 3.27 ± 2.51 |
| P-OP | t20:0 | 4.64 ± 4.79 | 5.85 ± 6.29 |
| P-OP | t21:0 | 2.83 ± 2.55 | 5.48 ± 6.39 |
| P-OP | t22:0 | 10.71 ± 5.9 | 16.47 ± 6.96 |
| P-OP | t23:0 | 0.40 ± 0.56 | 0.41 ± 0.58 |
| P-OP | t24:0 | n.d. | n.d. |
| P-OP | t25:0 | n.d. | n.d. |
| P-OP | t26:0 | n.d. | n.d. |
| P-OSD | d16:2 | n.d. | n.d. |
| P-OSD | d17:2 | n.d. | n.d. |
| P-OSD | d18:2 | 16.81 ± 9.12 | 85.05 ± 3.93 |
| P-OSD | d19:2 | n.d. | n.d. |
| P-OSD | d20:2 | 1.55 ± 0.79 | 7.67 ± 2.22 |
| P-OSD | d21:2 | 0.14 ± 0.11 | 0.74 ± 0.52 |
| P-OSD | d22:2 | 1.35 ± 0.89 | 6.49 ± 2.61 |
| P-OSD | d23:2 | 0.01 ± 0.01 | 0.07 ± 0.09 |
| P-OSD | d24:2 | n.d. | n.d. |
| P-OSD | d25:2 | n.d. | n.d. |
| P-OSD | d26:2 | n.d. | n.d. |

n.d., not detected.
